# Precise Shrink Fitting Design of the High Strength Gear Mold for the Precision Forging of Noncircular Spur Bevel Gears

**DOI:** 10.3390/ma16041556

**Published:** 2023-02-13

**Authors:** Wuhao Zhuang, Lin Hua, Xinghui Han, Wei Feng, Yanhui Wang, Mingzhang Chen

**Affiliations:** 1 School of Automotive Engineering, Wuhan University of Technology, Wuhan 430070, China; 2 Hubei Key Laboratory of Advanced Technology for Automotive Components, Wuhan University of Technology, Wuhan 430070, China; 3 Hubei Collaborative Innovation Center for Automotive Components Technology, Wuhan University of Technology, Wuhan 430070, China

**Keywords:** precision forging, shrink fitting, noncircular spur bevel gears, mold design, mold strength

## Abstract

Shrink fitting of forging mold (SFFM) is an effective method for improving mold strength, extending the mold’s service life and reducing the manufacturing cost of forging mold. However, due to the asymmetric geometry and complex stress distribution, the precise design of SFFM for the precision forging of noncircular bevel gears is very difficult. In this paper, a new precise design method of SFFM for the precision forging of noncircular bevel gears is proposed, which mainly includes the following five parts. First, a new design method for the mold parting surface—the curved surface parting method—is proposed to design the forging mold of noncircular spur bevel gears. Then, new dimension design methods for the gear mold and shrink rings based on the uniform shrinkage force are proposed. Third, a new design method for the inhomogeneous interference value between shrink rings and the gear mold is developed to provide a precise, uniform shrinkage force. After that, a strength correction method for the shrink-fitted gear mold is proposed to ensure the gear mold and shrink rings have sufficient strength both in the assembly process of the shrink-fitted gear mold and precision in the forging process of noncircular spur bevel gears. Ultimately, finite element simulations and verification experiments are performed to verify the proposed precise design method of SFFM for the precision forging of noncircular bevel gears. The precise design method of SFFM proposed in this paper is not only applicable to the precise design of the high-strength gear mold for noncircular bevel gears, but can also provide a valid reference for the precise design of the high-strength mold for other complicated asymmetric parts.

## 1. Introduction

Noncircular spur bevel gear is a type of gear that can realize variational speed ratio transmission between intersecting shafts [1]. It can execute the mutative speed ratio transmission, so as to perform many special transmission functions that cannot be performed by circular spur bevel gears (circular spur bevel gears can only execute the constant speed ratio transmission) [2]. Compared with the circular spur bevel gear, the shape of the noncircular spur bevel gear is more complex [3,4]. Due to the complicated geometry, the stress distribution in the gear mold during the precision forging of noncircular bevel gears is quite inhomogeneous, which causes the gear mold to be prone to failure. Therefore, it is a major objective to improve the strength and extend the service life of the gear mold for the forging mold design of noncircular bevel gears.

To improve the strength and extend the service life of the gear mold, an effective method can be taken by using several shrink rings to shrink the gear mold, which is called shrink fitting of forging mold (SFFM). In recent decades, design methods for SFFM have been researched by several researchers. These design methods can be mainly divided into two categories: the theoretical derivation method and the FE simulation method. Regarding the theoretical derivation method, Yeo et al. proposed a stress analysis procedure and design method for the shrink-fitted cold extrusion cylinder mold and introduced a theoretical derivation method of the contact pressure at the interface between the mold and shrink ring [5]. Qiu and Zhou provided an analytical method of calculating the contact pressure and stress distribution between multilayer, thick-walled cylinders with multicontact pairs and the temperature-raising effect [6]. Kutuk et al. designed the shrink-fitted mold of the precision gear forging by using both the analytical approach and the FE method [7]; the results indicated that the stress values obtained from the FE method were much higher than those obtained from the analytical approach, which meant that the thick-wall cylinder theory was not enough to guide the precision design of the shrink-fitted mold of the precision gear. Yilmaz [8] and Zuo [9], respectively, introduced the influence of the shrink fitting on the dimensional accuracy of precision forging dies for the solid cylinder and spur gear. As presented above, the theoretical derivation method is mainly suitable for the shrink fitting of the forging mold with the cylinder cavity, and there is a large deviation between the calculation results and actual results. Hence, several researchers tried to use the FE simulation method to analyze SFFM. Frater presented a FE simulation approach for analyzing the stress distribution of the shrink-fit forging mold for nonprismatic components [10]. Fu and Shang developed a boundary-element method program to analyze the stress distribution of the shrink-fit mold of a bevel gear [11]. Zhang et al. developed a method for designing interference fits in ring gear-wheel connections by using the FE simulation method, which can lead to more complete and accurate results than the traditional method of using the thick-wall cylinder theory [12]. Joun et al. presented an application-oriented finite-element approach for forging die structural analysis, and formulated the mold set structural analysis problem as a contact problem with both the shrink fit and the preloaded clamping [13]. Hur et al. compared the stiffness reinforcement of the shrink-fitted mold with different mold material configurations of the shrink ring and mold by using the FE simulation method, and the results showed that using sintered carbide as the material of the first stress ring can increase the stiffness of the shrink-fitted mold [14,15]. Eyercioglu et al. modified the analytical approach of the shrink-fitted mold of the precision gear forging based on FE simulation results, and several formulas and nomograms for mold design, shrink ring design and interference value design were provided [16]. Boutoutaou et al. researched the effect of the surface roughness on the stress distribution of the shrink fitting by using the FE analysis method [17]. Wang et al. proposed a method for determining the shrink-fitting ratio for the two-layer compound forging mold in the backward extrusion according to the FE simulation results [18]. Kamal et al. researched the effect of the radial thermal gradient on the stress distribution of multilayer, thick-walled cylinders [19]. In addition, several researchers investigated the influence of the shrink fitting on the performance of the forging mold. The integrated analysis for the shrink-fit, elastic deformation, fatigue, wear and brittle damage evolution of a cylindrical forging mold was presented by Yoh et al. [20], and the initial stress distribution due to the shrink fit of stress rings was defined. Chen et al. presented an optimal shrink fitting method with the least squares approach to minimize the deformation error of the axisymmetric closed-die forging mold [21]. Lee et al. investigated the effect of the shrink fitting ratio on the stress amplitude during the hexagonal bolt forging and gear extrusion, so as to control the level of the molded stress and increase the service life of these cold forging molds [22].

Despite the fact that several design methods for SFFM have been reported, these methods still have obvious limitations. The existing theoretical derivation and design method of SFFM is only appropriate for cylindrical-type parts whose forging mold can be equivalent to the thick-wall cylinder, but it is not suitable for designing SFFM for complex asymmetrical parts, such as noncircular bevel gears. Although the FE simulation method can design SFFM for complex asymmetrical parts, this method requires repeated trial and error in the process parameters, so as to obtain a relatively good shrink-fitting scheme. It means that the FE simulation design method of SFFM not only has low efficiency, but also has a limited optimization effect. Based on the above analysis, the existing design methods of SFFM are unlikely to be efficient and accurate enough for the precision forging of noncircular bevel gears. In this paper, combining the FE simulation method with the theoretical derivation method, a novel, precise design method of SFFM for the precision forging of noncircular bevel gears is proposed, which can significantly improve the efficiency and accuracy of shrink-fitting design of forging mold.

This paper includes five parts. In the first part, the mold parting surface for the forging mold of noncircular spur bevel gears is designed. In the second part, dimension design methods for the gear mold and shrink rings based on the uniform shrinkage force are developed. In the third part, a new design method for inhomogeneous interference values between shrink rings and gear molds is developed so as to provide a precise, uniform shrinkage force on the gear mold. In the fourth part, the strength correction method of shrink-fitted gear mold is proposed to ensure gear mold and shrink rings have sufficient strength both in assembling the process of shrink-fitted gear mold and the forging process of noncircular spur bevel gears. In the last part, several verification experiments are carried out to verify the effectiveness of the above methods.

## 2. The Design Method of the Mold Parting Surface for the Forging Mold of Noncircular Spur Bevel Gears

**Figure 1:**
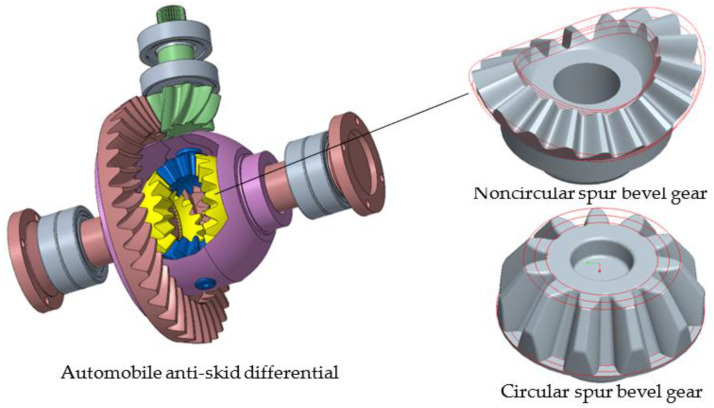
The automobile antiskid differential and the noncircular spur bevel gear.

The noncircular spur bevel gear studied in this paper is used in the antiskid differential of an automobile, as shown in Figure 1. Compared with the circular spur bevel gear, the shape of the noncircular spur bevel gear is more complex, which is mainly reflected in the following three aspects: (a) The teeth of the noncircular spur bevel gear do not meet the rotational symmetry relationship. (b) The pitch curve of the noncircular spur bevel gear is a spatially variable curvature curve. (c) There are obvious differences in the tooth surfaces between different teeth of the noncircular spur bevel gear, and the tooth surfaces on both sides of the same tooth are also different. The complex geometry of the noncircular spur bevel gear obviously increases the difficulty of the forging die design [23,24]. The design method of this noncircular spur bevel gear was developed by Zhuang [2], and the main design parameters are listed in Table 1.

**Figure 2:**
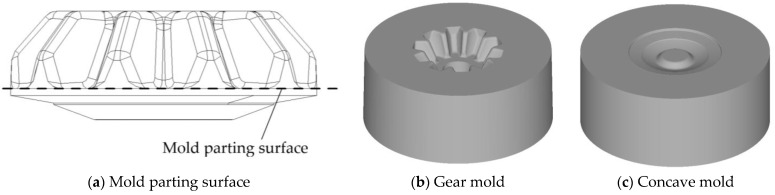
Plane parting method for mold parting surface of spur bevel gears.

**Figure 3:**
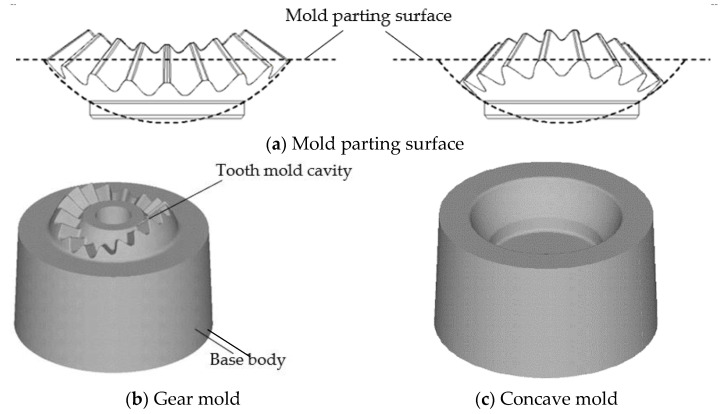
The design of the PPM forging mold for noncircular spur bevel gears.

**Figure 4:**
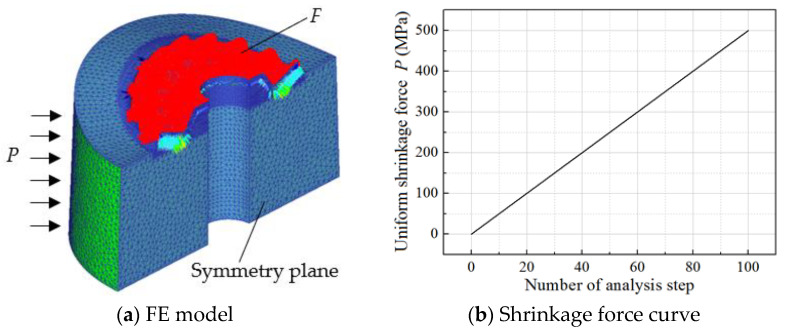
The FE model of equivalent stress distribution on the gear mold under shrinkage forces.

**Figure 5:**
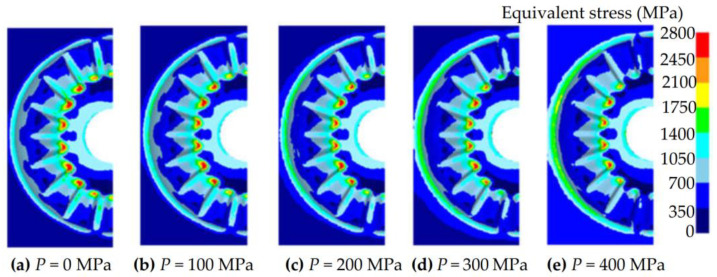
The equivalent stress distribution on the gear mold under different shrinkage forces.

In the design process of forging molds, the mold parting surface has a significant influence on the strength of the mold. The improper mold parting surface would lead to unreasonable stress distribution on the forging mold, thus, the strength of the forging mold has been significantly reduced. In the mold design of spur bevel gears, the largest radial cross-section of spur bevel gears is selected as the mold parting surface, as shown in Figure 2. This design method of the mold parting surface is defined as the plane parting method (PPM) in this research and has been widely applied in the forging mold design of spur bevel gears [25]. Different from spur bevel gears, the tooth shapes of noncircular bevel gears no longer satisfy rotational symmetry. If the PPM parting method is used to design the mold parting surface for noncircular bevel gears, the forging mold shown in Figure 3 would be obtained. As can be seen intuitively from Figure 3, the tooth mold cavity is obviously protruding from the base body of the gear mold, and the wall thickness of the tooth mold cavity is thin, which is easy to cause the cracking of the gear mold. In order to improve the strength of the gear mold, a shrinkage force is applied to the gear mold, and an FE model is developed to study the equivalent stress distribution on the gear mold under different shrinkage forces. The FE model is developed using Deform-3D V11 software, and the gear mold in this FE model is set as an elastic body and discrete into 150,000 mesh cells. The shrinkage force is loaded on the circumferential surface of the gear mold and the forging force is loaded on the surface of the gear mold cavity, as shown in Figure 4. By using this FE model, the equivalent stress distribution on the gear mold under different shrinkage forces is obtained, as shown in Figure 5. It can be seen from Figure 5 that with the increase in shrinkage force, the change in equivalent stress in the tooth mold cavity is very weak. This is because the shrinkage force is difficult to transfer to the protruding tooth mold cavity, and hardly enough to offset the tensile stress caused by forging force in the gear mold. To sum up, it is difficult to obtain a gear mold of noncircular bevel gears with sufficient strength by using the PPM parting method.

**Figure 6:**
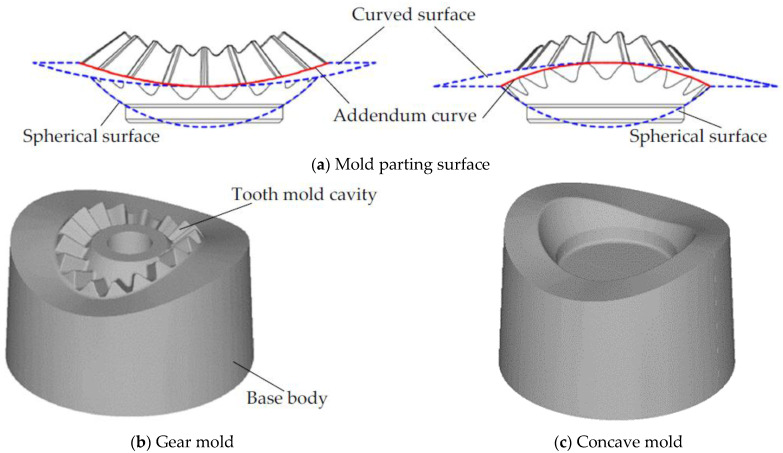
The design of the CSPM forging mold of noncircular bevel gears.

**Figure 7:**
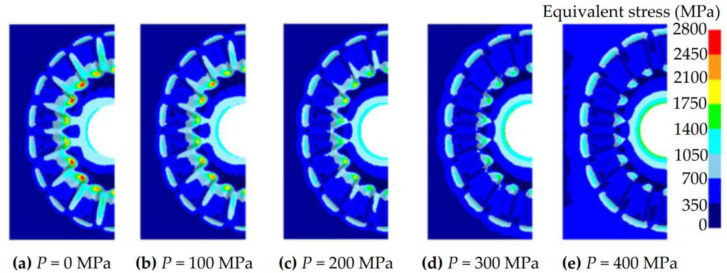
The equivalent stress distribution of the gear mold under different shrinkage forces.

According to the geometric characteristics of the noncircular spur bevel gear, a new mold parting surface design method, the curved surface parting method (CSPM), is proposed in this study. The principle of CSPM is that, by extending the addendum curve of the large gear-end of noncircular spur bevel gears radially outwards, a curved surface can be obtained as shown in Figure 6. Then, by merging the curved surface with the spherical surface of the large gear-end of noncircular bevel gears, the mold parting surface of noncircular bevel gears can be obtained, as shown in Figure 6. Compared with PPM, the tooth mold cavity designed by CSPM is embedded in the base body of the gear mold, and the cavity wall of the tooth mold cavity is thicker, so the strength of the gear mold can be increased. In order to illustrate the benefit of CSPM on the shrink-fitting design of the gear mold, a shrinkage force is applied to the gear mold designed by CSPM, and the equivalent stress distribution on the gear mold under different shrinkage forces shown in Figure 7 can be obtained by using the FE simulation method. Different from Figure 5, with the increase in the shrinkage force, the stress concentration area in the tooth mold cavity decreases significantly and the equivalent stress decreases rapidly, thus effectively reducing the risk of cracking the gear mold. This is because the tooth mold cavity of the gear mold designed by CSPM is submerged in the base body of the gear mold, and the shrinkage force can be transmitted to the tooth mold cavity through the base body, so the stress distribution state in the tooth mold cavity can be improved. In conclusion, compared with PPM, CSPM can not only increase the thickness of the tooth mold cavity wall, but also better exert the strengthening of shrink fitting of the gear mold, thereby this method can significantly improve the strength of the gear mold.

## 3. Dimension Design Methods of the Gear Mold and Shrink Rings Based on the Uniform Shrinkage Force

### 3.1. Accurate Prediction of the Forging Force

**Figure 8:**
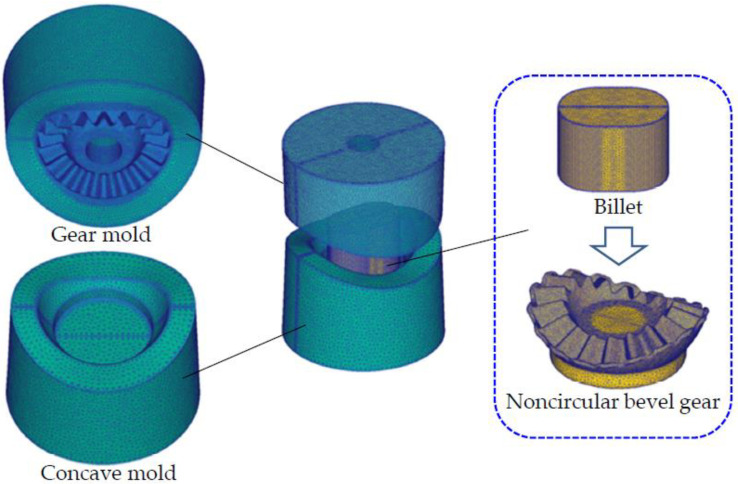
The FE model of the precision forging of a noncircular bevel gear.

**Figure 9:**
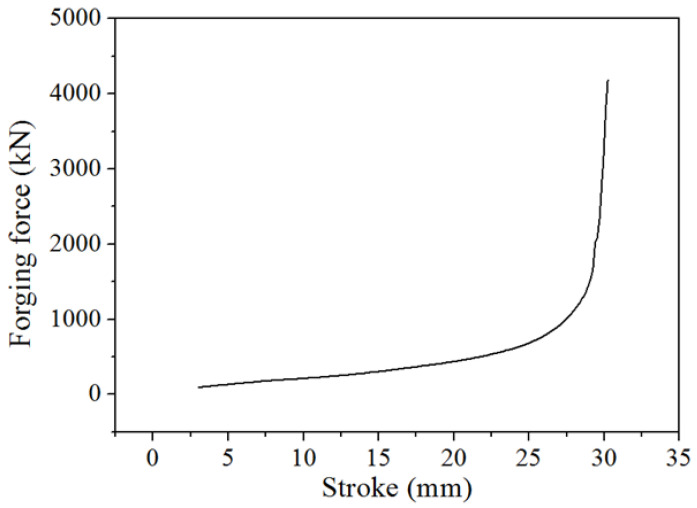
The forging force of the precision forging of the noncircular bevel gear.

To precisely design SFFM for the precision forging of noncircular bevel gears, the accurate forging force prediction of the precision forging of noncircular bevel gears is a basic precondition. The FE simulation method is currently the most accurate prediction method for the forging force; hence, a FE model for predicting the forging force of the precision forging of a noncircular bevel gear is developed under the Deform-3D platform, as shown in Figure 8. The FE model includes the gear mold, concave mold and billet; all of them are meshed using tetrahedral meshes, and the mesh numbers are listed in Table 2. The gear mold and concave mold are set as the rigid body, and the billet is set as the rigid plastic body. The material of the billet is 20CrMnTiH, and the constitutive modeling of 20CrMnTiH was developed by Feng and Wu [26,27]. The sheer friction model is applied to define the friction condition between forging molds and the billet, and heat transfers between forging molds and the billet are taken into consideration. The friction factor and heat transfer coefficient are listed in Table 2. The conjugate gradient iteration solver is used to calculate the plastic deformation and heat transfer in the precision forging of noncircular bevel gears. By using the FE model, the forging force of the precision forging of the noncircular bevel gear has been accurately predicted, as shown in Figure 9.

### 3.2. The Optimal Design Method for the Shrinkage Force and Gear Mold Dimension

As seen in Figure 7 in Section 2, applying a reasonable shrinkage force on the gear mold designed by the CSPM method can significantly improve the strength of the gear mold; how to accurately design the shrinkage force is a key issue in the shrink-fitting design of the gear mold. In addition, the shrinkage force required by the gear mold with different dimensions is different, so matching the design of the shrinkage force and the gear mold dimension should be carried out. In this part, an optimal design method for the shrinkage force and gear mold dimension based on the uniform shrinkage force is proposed. First, an FE model of the elastic deformation of the gear mold loaded by the forging force and shrinkage force is developed, as shown in Figure 4. In this FE model, the forging force *F* predicted in Section 3.1 is loaded on the surface of the gear mold cavity as a force boundary condition, and a shrinkage force *P* is added on the circumferential surface of the gear mold. To facilitate the optimal design process of the shrinkage force and gear mold dimension, the shrinkage force is assumed to be uniformly distributed. *P* is set as a function increasing linearly with analysis steps in the FE model (Figure 4b), so as to analyze the influence of *P* on the equivalent stress distribution of the gear mold. Since the noncircular bevel gear is plane-symmetric, it is enough to build half of the gear mold in the FE model, and the symmetry plane is set so that the efficiency and precision of the FE simulation can be improved.

**Figure 10:**
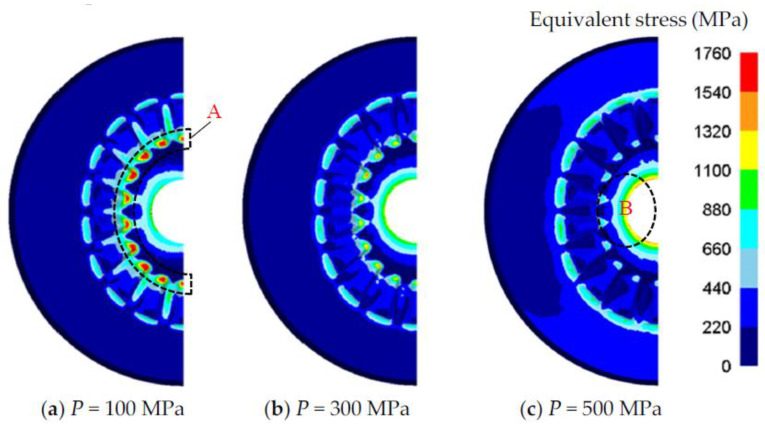
The evolution of the equivalent stress as *P* increases.

Figure 10 shows the equivalent stress distribution of the gear mold with increasing *P*. According to the equivalent stress evolution, the dangerous regions of the gear mold cavity can be determined. As can be seen in Figure 10a, the corner area between the bottom of the gear mold cavity and the gear-tooth cavity (region A) is a dangerous area of the gear mold when *P* is small. The reason is that the forging force *F* causes the tensile stress in the gear mold, and the tensile stress easily concentrates on region A due to the abrupt geometrical change. With the increase in *P*, the compression stress generated by *P* gradually cancels out the tensile stress generated by *F*, so the equivalent stress in region A gradually decreases, as shown in Figure 10b. When *P* is large enough, the edge of the inside hole (region B) becomes another dangerous area of the gear mold (Figure 10c). It is due to the fact that excessive *P* produces excessive compressive stress, and the compressive stress tends to concentrate near the inside hole region according to the thick-walled cylinder theory, so the equivalent stress becomes larger in region B. As long as the equivalent strain of region A and region B is less than the yield strength of the gear mold material $\sigma_{\mathrm{PD}}$, the gear mold has sufficient strength. It is easy to notice from the influence of *P* on the equivalent stress distribution of the gear mold, there is a minimum shrinkage force $P_{\min}$ to ensure that no failure occurs in region A and a maximum shrinkage force $P_{\max}$ to ensure that no failure occurs in region B.

**Figure 11:**
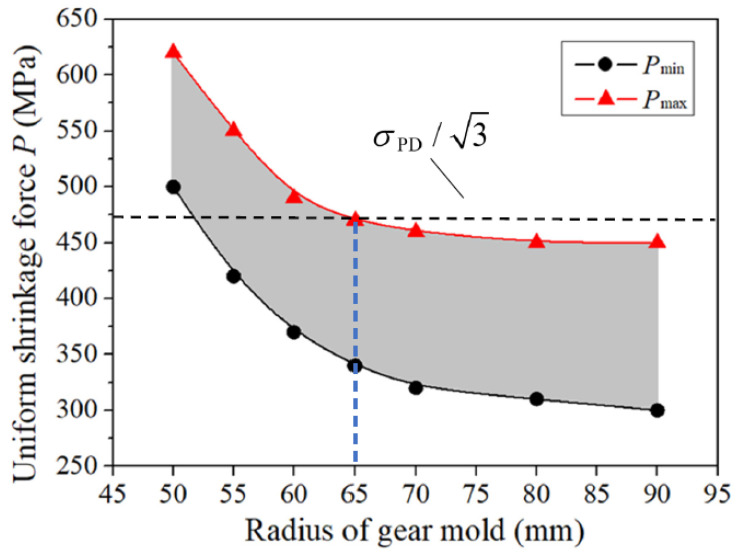
The evolving curve of $P_{\min}$ and $P_{\max}$ as *r*_1_ increases.

By using this FE model, the evolution of the equivalent stress distribution under different gear mold diameters *r*_1_ = {50, 55, 60, 65, 70, 80, 90} mm can be investigated, and evolving curves of $P_{\min}$ and $P_{\max}$ with increasing * r*_1_ can be obtained, as shown in Figure 11. The material of the gear mold is H13 steel, and the allowable stress of H13 steel is $\sigma_{PD}=1420\;\mathrm{MPa}$ . As can be seen in Figure 11, both $P_{\min}$ and $P_{\max}$ gradually decrease as *r*_1_ increases, it is due to the fact that the cavity wall of the gear model becomes thicker and the strength of the gear mold increases, so the shrinkage force requirement decreases. When *r*_1_ > 65 mm, both evolving curves of $P_{\min}$ and $P_{\max}$ tend to be stable, which means that the effect of *r*_1_ on $P_{\min}$ and $P_{\max}$ becomes weak. It can be seen from Figure 11, a reasonable selection range of *P* and *r*_1_ is determined, which can provide a basis for the optimal design of the shrinkage force and gear mold dimension.

### 3.3. The Optimal Dimension Design Method of Shrink Rings

Since the shrinkage force *P* between the gear mold and shrink ring is assumed as the uniformly force in Section 3.2, the loading status of shrink rings can be equivalent to thick-walled cylinders bearing with a uniform internal pressure, so the circumferential stress $\sigma_{t}$, radial stress $\sigma_{r}$ and axial stress $\sigma_{z}$ of any point in the shrink ring can be calculated by using Lame formula [28]. Equation (1) is the formulas of $\sigma_{t}$, $\sigma_{r}$ and $\sigma_{z}$, and the equivalent stress $\bar{\sigma }$ can be computed by Equation (2).

(1) $$\left\{ \begin{array}{l} \sigma_{t}=\frac{r_{1}^{2}p_{1}-r_{2}^{2}p_{2}}{r_{2}^{2}-r_{1}^{2}}+\frac{(p_{1}-p_{2})r_{1}^{2}r_{2}^{2}}{r^{2}(r_{2}^{2}-r_{1}^{2})}\\ \sigma_{r}=\frac{r_{1}^{2}p_{1}-r_{2}^{2}p_{2}}{r_{2}^{2}-r_{1}^{2}}-\frac{(p_{1}-p_{2})r_{1}^{2}r_{2}^{2}}{r^{2}(r_{2}^{2}-r_{1}^{2})}\\ \sigma_{z}=0 \end{array}\right.$$

(2) $$\bar{\sigma }=\sqrt{\sigma_{t}^{2}(r_{1})+\sigma_{r}^{2}(r_{1})-\sigma_{t}^{}(r_{1})\sigma_{r}^{}(r_{1})}=\sqrt{{\left(\frac{r_{1}^{2}p_{1}-r_{2}^{2}p_{2}}{r_{2}^{2}-r_{1}^{2}}\right)}^{2}+3{\left(\frac{(p_{1}-p_{2})r_{1}^{2}r_{2}^{2}}{r^{2}(r_{2}^{2}-r_{1}^{2})}\right)}^{2}}$$

where, r is the radius of any point in the shrink ring, $r_{1}$ is the radius of the gear mold, which equal to the inner radius of the shrink ring, $r_{2}$ is the outer radius of the shrink ring, $p_{1}$ and $p_{2}$ are the pressure on the inner surface and outer surface of the shrink ring respectively.

It is easy to notice from Equations (1) and (2) that the small r is, the larger $\bar{\sigma }$ is, which means that the inner surface of the shrink ring is most vulnerable to be yielded according to Von Mesis criterion. Hence, as long as the equivalent stress $\bar{\sigma }$ on the inner surface of the shrink ring is less than the allowable stress of the shrink ring material $\sigma_{\mathrm{PR}}$, the strength of the shrink ring is sufficient. In the shrink fitting design of the forging mold, more layers of the shrink ring would obviously increase the complexity of the shrink fitting process and mold manufacturing costs, so one or two layers of the shrink ring are frequently used to shrink fit the forging mold. Based on the above analysis, dimension design methods for the single-layer shrink fitting scheme (S-L scheme) and double-layer shrink fitting scheme (D-L scheme) are developed as follows.

(1) Single-layer shrink fitting scheme

**Figure 12:**
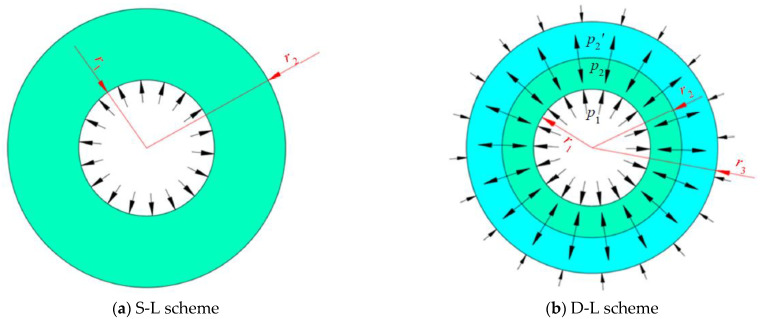
Loading status of shrink rings.

In the S-L scheme, the pressures on the inner surface and outer surface of shrink ring are $p_{1}=-P$ and $p_{2}=0\;\mathrm{MPa}$ respectively, and the loading status of the shrink ring is shown in Figure 12a. Substituting $p_{1}=-P$ , $p_{2}=0\;\mathrm{MPa}$ to Equation (2), the equivalent stress on the inner surface of the shrink ring can be calculated:

(3) $$\bar{\sigma }\left(r_{1}\right)=\sqrt{{\left(\frac{r_{1}^{2}P}{r_{2}^{2}-r_{1}^{2}}\right)}^{2}+3{\left(\frac{r_{1}^{2}r_{2}^{2}P}{r^{2}(r_{2}^{2}-r_{1}^{2})}\right)}^{2}}$$

According to Von Mesis yield criterion, as long as $\bar{\sigma }\left(r_{1}\right)=\sigma_{\mathrm{PR}}$, the optimum radius of the shrink ring can be obtained to meet the strength of the shrink ring, so the relationship of $r_{1}$ and $r_{2}$ can be obtained, as shown in Equation (4).

(4) $$r_{2}=\frac{r_{1}\sqrt{\sqrt{4\sigma_{\mathrm{PR}}^{2}-3P^{2}}+P}}{\sqrt{\sqrt{4\sigma_{\mathrm{PR}}^{2}-3P^{2}}-3P}}$$

To ensure Equation (4) valid, *P* has to meet Equation (5).

(5) $$P\leq \sigma_{\mathrm{PR}}/\sqrt{3}$$

(2) Double-layer shrink fitting scheme

In the D-L scheme, two shrink rings, inside ring and outside ring, are used to shrink fit the gear mold, and the loading statuses of the inside ring and outside ring are shown in Figure 12b. The pressures on the inner surface and outer surface of inside ring are $p_{1}=-P$ and $p_{2}$, and the pressures on the inner surface and outer surface of the outer ring are ${p^{\prime }}_{2}=-p_{2}$ and $p_{3}=0\;\mathrm{MPa}$. Plugging these loading statuses to Equation (1), the stress state on the inner surface of the inside ring and outside ring can be calculated by using Equations (6) and (7).

(6) $$\left\{ \begin{array}{l} \sigma_{t}(r_{1})=\frac{(r_{1}^{2}+r_{2}^{2})P}{r_{1}^{2}-r_{2}^{2}}-\frac{2r_{2}^{2}p_{2}}{r_{2}^{2}-r_{1}^{2}}\\ \sigma_{r}(r_{1})=P \end{array}\right.$$

(7) $$\left\{ \begin{array}{l} \sigma_{t}(r_{2})=\frac{(r_{1}^{2}+r_{2}^{2})p_{2}}{r_{1}^{2}-r_{2}^{2}}\\ \sigma_{r}(r_{2})=-p_{2} \end{array}\right.$$

In this scheme, Tresca yield criterion Equation (8) is adopted as the failure criteria of shrink rings, which can obviously reduce the complexity of the formula derivation than Von mesis yield criterion.

(8) $$\sigma_{t}-\sigma_{r}=\sigma_{\mathrm{PR}}$$

Substituting Equations (6) and (7) to Equation (8), the relationship of $r_{1}$, $r_{2}$ and $r_{3}$ can be obtained, as shown in Equations (9) and (10).

(9) $$r_{2}=\frac{r_{1}\sqrt{\sigma_{\mathrm{PR}}}}{\sqrt{\sigma_{\mathrm{PR}}-P}}$$

(10) $$r_{3}=\frac{r_{1}\sigma_{\mathrm{PR}}}{\sigma_{\mathrm{PR}}-P}$$

To ensure Equations (9) and (10) valid, *P* has to meet Equation (11) in this scheme.

(11) $$P\leq \sigma_{\mathrm{PR}}$$

**Figure 13:**
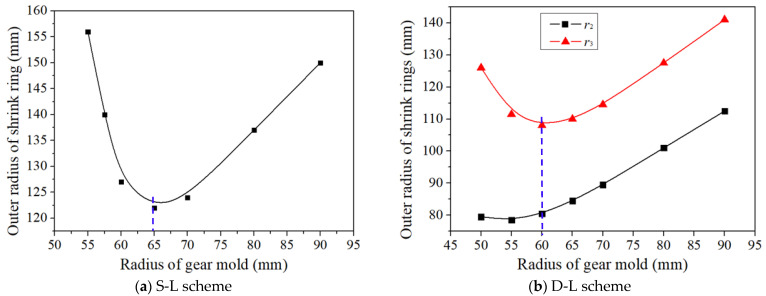
The design curve of the radius of shrink rings.

It is not difficult to understand that larger *P* on the inner surface of shrink rings require larger shrink rings to meet the strength requirement of shrink rings. Hence, in order to reduce the size of shrink rings, the shrink ring must be designed according to $P_{\min}$ curve which has been obtained in Figure 11. In the case of SFFM for the precision forging of noncircular bevel gears investigated in this research, the material of shrink ring is steel AISI-4140, and the allowable stress of this material is $\sigma_{\mathrm{PR}}=\;833\;\mathrm{MPa}$. As can be seen from Figure 11, when $r_{1}>52\;\mathrm{mm}$, $P_{\min}<\sigma_{\mathrm{PR}}/\sqrt{3}$, so S-L scheme can be adopted for SFFM. Figure 13a is the design curve of $r_{2}$ by using S-L scheme. As can be seen from Figure 13a, as radius of gear mold $r_{1}$ increases, $r_{2}$ decreases firstly and then increases. The reason for this phenomenon is that *P* is quite large when $r_{1}$ is very small (as shown in Figure 11), so it is necessary for a large thickness shrink ring to ensure the strength of the shrink ring. As $r_{1}$ increases, *P* obviously decreases, so the stress in the gear mold reduces, and the thinner shrink ring can also ensure the gear mold has the sufficient strength. Although the influence of $r_{1}$ on *P* is weak when $r_{1}\geq \;65\;\mathrm{mm}$, $r_{2}$ still steadily increases as $r_{1}$ increases according to Equation (4). It can be seen from Figure 13a, the minimum value of $r_{2}$ is 122 mm when $r_{1}=\;65\;\mathrm{mm}$, which is the optimal radius of the shrink ring for the S-L scheme (listed in Table 3).

It can been seen from Figure 11, the whole $P_{\min}$ in $5\mathrm{mm}\;<r_{1}<90\mathrm{mm}$ are smaller than $\sigma_{\mathrm{PR}}$, which meets Equation (11), so the D-L scheme is also can be used to shrink fit this gear mold. Figure 13b shows the design curve of $r_{2}$ and $r_{3}$ with increasing $r_{1}$ calculated by Equations (9) and (10). As can be seen in Figure 13b, both $r_{2}$ and $r_{3}$ also decrease firstly and then increase. The reason of this phenomenon is similar to the S-L scheme. Since the maximum radius of the shrink fitted gear mold depends on the maximum radius of the outside ring $r_{3}$ , the optimal radiuses of shrink rings for D-L scheme can be determined when $r_{3}$ is minimum. The optimal radiuses of shrink rings by using the D-L scheme are listed in Table 3.

As seen in Table 3, the maximum radius of the shrink-fitted gear mold by using S-L scheme is 122 mm, which is only 14 mm larger than the maximum radius of the shrink-fitted gear mold by using the D-L scheme. It means that there is little difference in the gear mold size between the S-L scheme and the D-L scheme in this case. However, compared with the D-L scheme, the S-L scheme can obviously reduce the interference assembly difficulty and machining cost of shrink rings, therefore, the S-L shrink ring scheme is the optimal scheme in this case.

## 4. The Design Method of Inhomogeneous Interference Values between Shrink Rings and the Gear Mold

**Figure 14:**
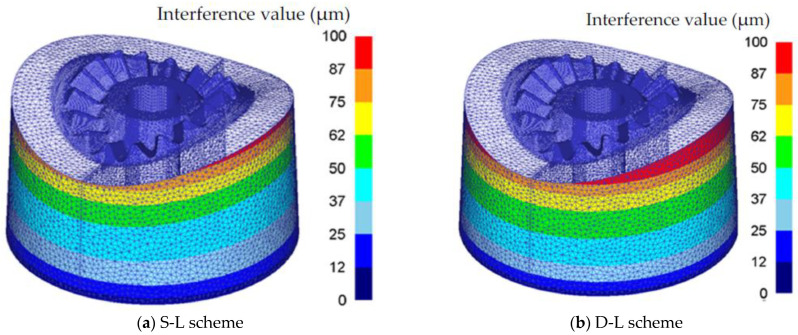
The distribution of $\Delta r_{1}^{\mathrm{die}}$ on the outer surface of the gear mold.

The shrinkage force is generated by the interference assembly between the shrink ring and gear mold, so the interference value between the shrink ring and gear mold should be accurately designed to produce the appropriate shrinkage force. The inhomogeneous interference values between shrink rings and the gear mold consist of two parts: elastic deformation on the outer surface of gear mold and elastic deformation on the inner surface of the shrink rings. Loaded by the forging force and shrinkage force, the radius of the gear mold decreases $\Delta r_{1}^{\mathrm{die}}$, the inner radius of the shrink ring increases $\Delta r_{1}^{\mathrm{ring}}$, and the sum of $\Delta r_{1}^{\mathrm{die}}$ and $\Delta r_{1}^{\mathrm{ring}}$ is the interference value $\Delta r_{1}$ between the shrink ring and gear mold. Since the elastic deformation of the gear mold loaded by the forging force and shrinkage force can be simulated by using the FE model developed in Section 3.2, $\Delta r_{1}^{\mathrm{die}}$ can be obtained from the FE simulation result by measuring the radial displacement on the circumferential surface of the gear mold, as shown in Figure 14. $\Delta r_{1}^{\mathrm{ring}}$ is the radial displacement on the inner surface of the shrink ring, which can be derived according to the Lame formula and Hooke’s law, and the calculation equation of $\Delta r_{1}^{\mathrm{ring}}$ is given as Equation (12).

(12) $$\Delta r_{1}^{\mathrm{ring}}=\{\begin{array}{cc} \frac{r_{1}P}{2E}\left(\frac{r_{2}^{2}+r_{1}^{2}}{r_{2}^{2}-r_{1}^{2}}+\mu \right) & \;(S-L\;\mathrm{scheme})\\ \frac{r_{1}r_{3}^{2}\left(4P-\sigma_{\mathrm{PR}}\right)+r_{1}^{3}\left(2P+\sigma_{\mathrm{PR}}\right)}{2E\left(r_{3}^{2}-r_{1}^{2}\right)}+\mu \frac{r_{1}P}{E} & \;(D-L\;\mathrm{scheme}) \end{array}$$

where, E is the Young modulus of the shrink ring material, μ is the Poisson’s ratio of the shrink ring material.

In addition, in the D-L scheme, there is another interference value $\Delta r_{2}$ between the inside ring and outside ring, which can be calculated by Equation (13).

(13) $$\Delta r_{2}^{}=\frac{2Pr_{2}r_{3}^{2}-\sigma_{\mathrm{PR}}r_{2}\left(r_{3}^{2}-r_{1}^{2}\right)}{2E(r_{3}^{2}-r_{2}^{2})}$$

Substituting $r_{1}$, $r_{2}$, $r_{3}$ and *P* listed in Table 3 to Equations (12) and (13), interference values ($\Delta r_{1}{}^{\mathrm{ring}}$ and $\Delta r_{2}$) can be calculated by using the S-L scheme and D-L scheme, as listed in Table 3. Adding $\Delta r_{1}{}^{\mathrm{ring}}$ to $\Delta r_{1}^{\mathrm{die}}$, the inhomogeneous interference value $\Delta r_{1}$ between the shrink ring and gear mold can be obtained.

## 5. The Strength Correction Method of the Shrink-Fitted Gear Mold

**Figure 15:**
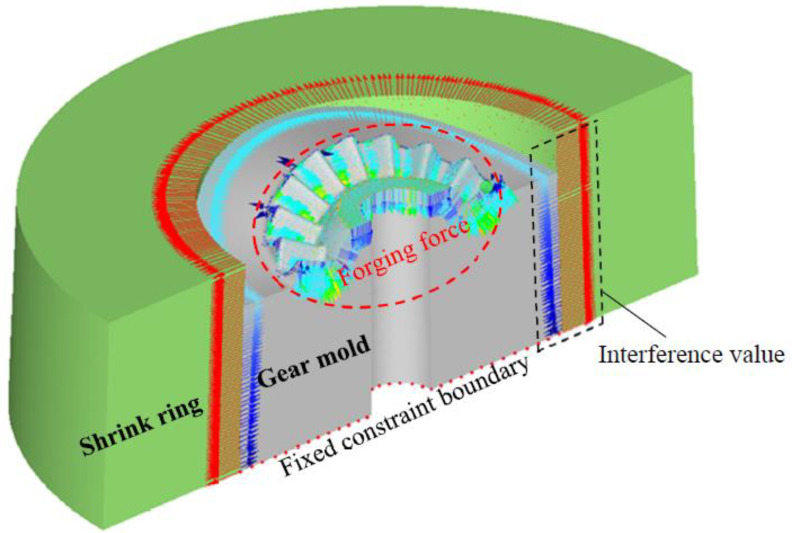
The strength correction FE model of the shrink fitted gear mold.

**Figure 16:**
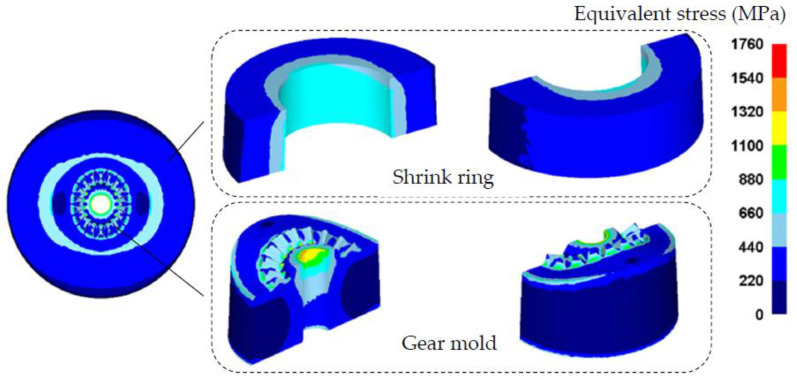
The equivalent stress distribution on the shrink-fitted gear mold in the forging process of the noncircular bevel gear.

In the above research, key optimal SFFM parameters for the precision forging of noncircular bevel gear have been designed, and these parameters should be verified to ensure that the shrink-fitted gear mold has sufficient strength. According to the optimal SFFM parameters of the S-L scheme listed in Table 3, a strength correction FE model of the shrink-fitted gear mold is developed, as shown in Figure 15. The FE model consists of a gear mold and a single-layer shrink ring, the forging force *F* is obtained in Section 3.1 is loaded on the mold cavity surface of the gear mold, and the inhomogeneous interference value $\Delta r_{1}$ calculated in Section 4 is added between the shrink ring and gear mold. A fixed constraint boundary is set on the bottom of the gear mold so as to avoid the rigid displacement of the gear mold, and only half of the gear mold and shrink ring is developed in the FE mold so as to improve the efficiency and precision of the FE simulation. By using the FE model, the equivalent stress distribution on the shrink ring and gear mold during the forging process of the noncircular bevel gear (both the forging force and shrinkage force loaded on the gear mold) is obtained, as shown in Figure 16. It can be seen from Figure 16, the equivalent stresses on the gear mold and shrink ring are all smaller than the allowance stress of materials, which means that the S-L scheme designed above can ensure the shrink fitted gear mold has the sufficient strength.

**Figure 17:**
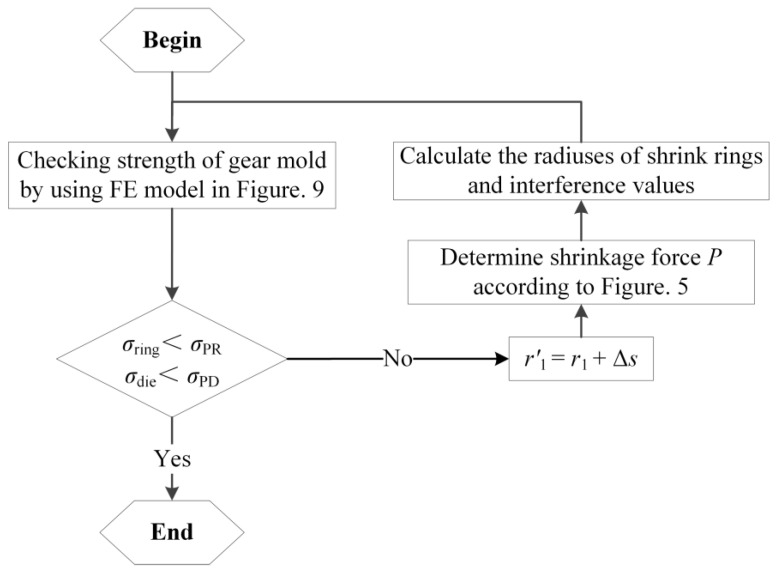
The route diagram of the strength correction of the shrink-fitted gear mold.

**Figure 18:**
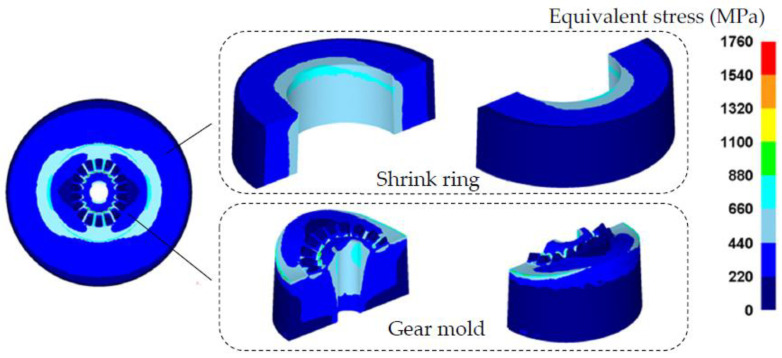
The equivalent stress distribution of the shrink-fitted gear mold in the shrink-fitting process.

The shrink fitting scheme designed above is to ensure the strength of the gear mold during forging process (loaded by both the forging force and shrinkage force). However, it is also necessary to ensure that both the gear mold and shrink rings are not failed in the assembling process (only the shrinkage force *P* is loaded on the gear mold), hence the strengths of the gear mold and shrink ring in the assembling process also need to be checked. Based on the FE model in Figure 15, the forging force on the mold cavity surface of the gear mold should be removed, so that the equivalent stress distribution on the shrink ring and gear mold in the assembling process can be obtained. If the equivalent stresses of the gear mold and shrink ring are smaller than the allowance stress of the mold material, the shrink fitting scheme is effective. Otherwise, the shrink fitting scheme should be corrected by increasing the radius of the gear mold $r_{1}$. The correction process is shown in Figure 17, and the specific operation steps are presented as follows: increasing $r_{1}$ by Δs to $r_{1}{}^{\prime }$, determining the shrinkage force *P* according to Figure 11, calculating the radiuses of shrink rings and interference values by design methods proposed in Section 3.3 and Section 4, and then checking the strengths of the gear mold and shrink rings by using the FE model in Figure 15. If the strengths of the gear mold and shrink rings meet strength requirements, the correction process is complete. Otherwise, repeating the correction process until the strengths of the gear mold and shrink rings meet strength requirements. Figure 18 shows the equivalent stress distribution on the gear mold in the assembling process before the correction. It is easy to notice that the equivalent stresses on the gear mold and shrink ring are all smaller than the allowance stress of the materials, so it is unnecessary for the shrink-fitting scheme in the research to be corrected.

## 6. Verification Experiments

**Figure 19:**
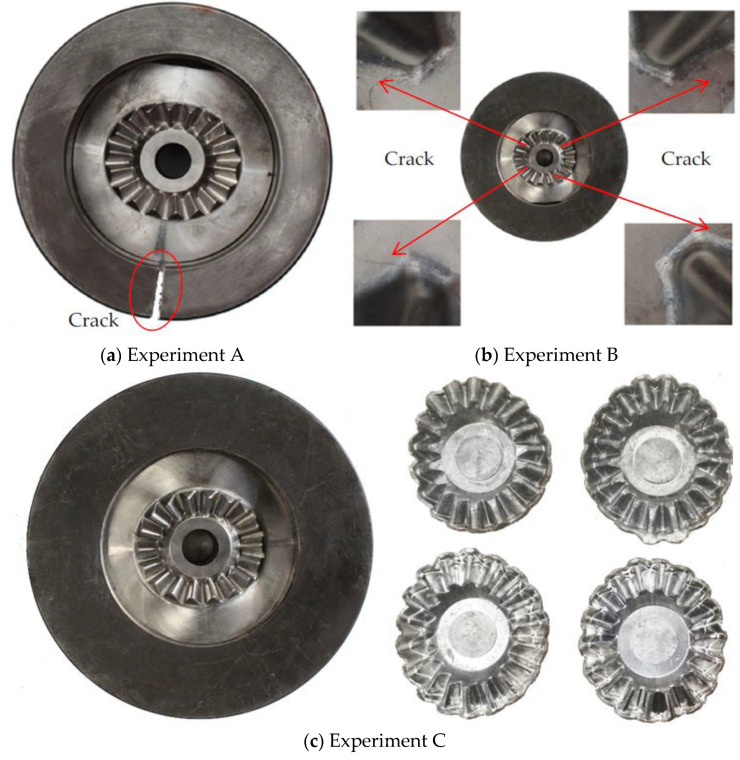
Experiment results of three verification experiments.

To verify the effectiveness of the precise design method of SFFM for the precision forging of noncircular bevel gears proposed in this paper, three verification experiments were performed in this research, and key parameters of these verification experiments are listed in Table 4. In experiment A, only the radius of the shrink ring decreased to 200 mm based on the S-L scheme in Table 3. In experiment B, only $\Delta r_{1}^{\mathrm{ring}}$ dropped from 0.257 mm to 0.200 mm based on the S-L scheme. Experiment C was designed according to the SFFM parameters of the S-L scheme in Table 3. Figure 19 shows the experimental results of three verification experiments. As can be seen in Figure 19a, the shrink ring is cracked in experiment A. The reason is that the strength of the shrink ring decreased due to the reduction in the radius of the shrink ring, so that the shrink ring cannot sustain the strong tensile stress caused by the forging force. In Figure 19b, many cracks appear at the bottom of the gear mold cavity, which indicates that the strength of the gear mold in experiment B is insufficient. It is due to the fact that the shrink ring cannot provide sufficient shrinkage force to the gear mold because of the smaller interference value between the shrink ring and gear mold. In Figure 19c, no cracks appear on both the gear mold and shrink ring in experiment C, which indicates that the S-L scheme design in the research (listed in Table 3) can ensure the sufficient strength of the shrink ring and gear mold. These experiment results effectively verify the reliability of the design method of SFFM for the precision forging of noncircular bevel gears proposed in this paper.

## 7. Conclusions

This research proposes a precise shrink-fitting design method for the high-strength gear mold for the precision forging of noncircular spur bevel gears. Several key design problems of the shrink-fitting design method are solved by combining the FE simulation method with the theoretical derivation method, and some conclusions are listed as follows.

1. A new mold parting surface design method, the curved surface parting method (CSPM), is proposed, which can not only increase the thickness of the tooth mold cavity wall but also better exert the strengthening of shrink fitting of the gear mold, thereby this method can significantly improve the strength of the gear mold.
2. Based on the stress distribution of the gear mold, the nonlinear relationship between the uniform shrinkage force *P* and the radius of the gear mold *r*_1_ is established. Both the minimum shrinkage force $P_{\min}$ and the maximum shrinkage force $P_{\max}$ gradually decrease as *r*_1_ increases. On this basis, the reasonable selection range of *P* and *r*_1_ is obtained, and an optimal design method of the shrinkage force and gear mold dimension is proposed.
3. With the radius of the gear mold increasing, the outer radius of shrink rings first decreases and then increases, and an optimal dimension of the shrink rings for the S-L scheme and the D-L scheme is developed according to the design curve of the radius of shrink rings.
4. The application conditions for the S-L scheme and the D-L scheme are discussed, i.e., the S-L scheme is suitable when $0\leq P<\sigma_{\mathrm{PR}}/\sqrt{3}$, and the D-L scheme is suitable when $P<\sigma_{\mathrm{PR}}$.
5. The inhomogeneous interference values between shrink rings and the gear mold consist of two parts: elastic deformation on the outer surface of the gear mold and elastic deformation on the inner surface of shrink rings. The elastic deformation of the gear mold can be obtained from the FE simulation result, and the elastic deformation of shrink rings can be derived according to the Lame formula and Hooke’s law.
6. Three verification experiments were performed to verify the effectiveness of the precise design method of SFFM for the precision forging of noncircular bevel gears. When the radius of the shrink ring was smaller than the design value, cracks appeared on the shrink ring. When the interference value was smaller than the design value, cracks appeared on the gear mold. No crack appeared when the parameters of SFFM were set to design value.

## Figures and Tables

**Table 1:** Main design parameters of the noncircular bevel gear.

Parameters	Value
Pressure angle (°)	25
Tooth number	18
Mean Module (mm)	3.735
gear ratio function	g(φ_i_) = −1/[0.9487 × cos(φi) + 2.2137]
Mean cone distance (mm)	37
Face width (mm)	18

**Table 2:** Main forging parameters of the precision forging of the noncircular bevel gear.

Parameters	Value
Temperature of billet (°C)	950
Preheating temperature of mold(°C)	250
Friction factor	0.3
Heat transfer coefficient (kW/m^2^·°C)	11
Feed speed of upper mold (mm/s)	15
Mesh number of billet	15,000
Mesh number of gear mold	12,000
Mesh number of concave mold	80,000

**Table 3:** Optimal SFFM parameters for the noncircular bevel gear.

	*P* (MPa)	*r*_1_ (mm)	*r*_2_ (mm)	*r*_3_ (mm)	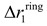 (mm)	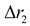 (mm)
S-L scheme	340	65	122	/	0.257	/
D-L scheme	370	60	80.5	108	0.270	0.331

**Table 4:** Key parameters and results of verification experiments.

	*r*_1_ (mm)	*r*_2_ (mm)	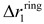 (mm)	Results
Experiment A	65	100	0.257	Cracked in shrink ring
Experiment B	65	122	0.200	Cracked in gear mold
Experiment C	65	122	0.257	No cracks

## Data Availability

The data presented in this study are available within the article.
